# Precursor Concentration-Dependent Sol–Gel Dynamics in Neodymium Oxide: From Gel Framework to Electrochemical Functionality in Asymmetric Supercapacitors

**DOI:** 10.3390/gels11110883

**Published:** 2025-11-03

**Authors:** Rutuja U. Amate, Aditya A. Patil, Aviraj M. Teli, Sonali A. Beknalkar, Chan-Wook Jeon

**Affiliations:** 1 School of Chemical Engineering, Yeungnam University, 280 Daehak-ro, Gyeongsan 712-749, Republic of Koreaaditya.nanotechnology@gmail.com (A.A.P.); 2 Division of Electronics and Electrical Engineering, Dongguk University-Seoul, Seoul 04620, Republic of Korea

**Keywords:** rare earth oxide, sol–gel synthesis, precursor concentration, hierarchical morphology, pseudocapacitance, asymmetric supercapacitor

## Abstract

Rare-earth oxides possess distinctive electronic configurations, tunable oxidation states, and inherent structural robustness, making them highly attractive for advanced energy storage applications. Among these, neodymium oxide (Nd_2_O_3_) stands out due to its high surface redox activity, structural stability, and favorable band alignment, enabling efficient charge storage in electrochemical devices. In this study, Nd_2_O_3_ electrodes were synthesized via a sol–gel method with systematically varied precursor concentrations (1 mM, 3 mM, and 5 mM) to elucidate the impact of synthesis on crystallinity, morphology, and electrochemical performance. X-ray diffraction (XRD) confirmed the formation of the hexagonal Nd_2_O_3_ phase, with the 3 mM sample (Nd-2) exhibiting the sharpest reflections, indicative of enhanced crystallinity and reduced lattice defects. X-ray photoelectron spectroscopy (XPS) revealed trivalent Nd species and both lattice and surface oxygen, providing abundant redox-active sites. Field Emission Scanning Electron Microscope (FE-SEM) showed Nd-2 possessed a hierarchically interconnected fibrous network decorated with fine granules, maximizing active surface area and facilitating rapid ion diffusion. Electrochemical testing demonstrated that Nd-2 achieved an areal capacitance of 20 F cm^−2^, a diffusion-controlled pseudocapacitive contribution of ~84.9%, and retained 86.3% capacitance over 12,000 cycles. An asymmetric supercapacitor with Nd-2 and activated carbon delivered an energy density of 0.132 mWh cm^−2^, power density of 1.8 mW/cm^2^, and 81.1% capacitance retention over 7000 cycles. These results highlight the critical role of precursor concentration in tailoring structure and electrochemical performance, establishing Nd_2_O_3_ as a promising electrode for high-performance energy storage devices.

## 1. Introduction

In nature, the delicate balance between abundance and scarcity dictates the efficiency of fundamental processes. For example, the thin ozone layer, although present in only trace concentrations, performs the irreplaceable role of protecting the biosphere from harmful ultraviolet radiation [1]. This paradox of minute presence yet profound function parallels the challenge in designing advanced energy storage devices, where even small modifications in material composition or structure can dramatically alter their performance. Within the rapidly evolving field of electrochemical energy storage, supercapacitors have emerged as indispensable components because they bridge the gap between conventional capacitors, which exhibit high power but low energy density, and batteries, which provide high energy density but relatively slow charge–discharge characteristics [2]. As a result, supercapacitors have attracted widespread attention in applications ranging from portable electronics to electric vehicles and renewable energy storage systems. Nevertheless, their overall energy density remains constrained by the intrinsic limitations of electrode materials, which must simultaneously exhibit high surface area, superior conductivity, and robust structural stability under extended cycling [3,4,5,6]. Over the past decade, extensive research efforts have focused on exploring diverse categories of electrode materials. Transition metal oxides such as MnO_2_, NiCo_2_O_4_, Fe_2_O_3_, and WO_3_ have been intensively studied due to their rich redox chemistry and ability to deliver high pseudocapacitance [7,8,9]. Conducting polymers including polyaniline and polypyrrole offer high theoretical capacitance and facile charge storage mechanisms but often suffer from severe volumetric expansion and poor cycle stability. Carbon-based materials such as activated carbon, graphene, and carbon nanotubes remain attractive due to their excellent electrical conductivity and long cycle life, yet their low specific capacitance continues to limit their practical energy density [10,11,12]. These challenges have motivated the search for alternative materials that combine the advantageous features of each category while mitigating their inherent weaknesses. Rare-earth oxides have recently emerged as highly promising candidates because of their unique 4f-electronic configurations, tunable oxidation states, defect-rich structures, and excellent thermal and chemical stability [4,13,14,15,16]. In particular, Nd_2_O_3_ offers favorable band alignment, high surface redox activity, and structural robustness, making it a strong contender for asymmetric supercapacitor systems, where an extended voltage window and enhanced energy density can be achieved without compromising cycle life [17,18,19,20].

Several research groups have highlighted the potential of Nd_2_O_3_-based materials for supercapacitor applications. Kubra et al. synthesized Nd_2_O_3_/Mn_3_O_4_ nanocomposites through hydrothermal processing, reporting that the synergistic interaction between Nd_2_O_3_ and Mn_3_O_4_ improved pseudocapacitive behavior, with the optimal composite achieving a capacitance of 205.29 F g^−^^1^ and high coulombic efficiency [21]. Imtiaz et al. demonstrated that Nd-doping significantly enhanced the performance of SmFeO_3_, raising the capacitance to 1455.9 F g^−^^1^ and improving cyclic retention, thus confirming the role of Nd in boosting charge transfer kinetics [22]. Munawar et al. fabricated Nd_2_CeMO_3_ (M = Er, Sm, V) nanostructures via sol–gel synthesis, where co-doping strategies enhanced conductivity and electrochemical activity, with Nd_2_CeErO_3_ exhibiting 1319 F g^−^^1^ along with superb stability [23]. Shiri et al. reported Nd_2_O_3_ nanorods obtained by ultrasound-assisted electrochemical deposition and their hybridization with POAP, which led to significantly improved capacitance (379 F g^−^^1^) and excellent rate capability [24]. Li et al. prepared PANI/Nd_2_O_3_ composites by oxidative polymerization, where the incorporation of Nd_2_O_3_ reduced charge-transfer resistance and enhanced pseudocapacitance, although excessive oxide loading decreased conductivity [25]. Collectively, these works underscore the promising role of Nd_2_O_3_ in advanced supercapacitor technologies, while simultaneously exposing challenges such as complex processing requirements, difficulties in morphological control, and limited scalability associated with hydrothermal, electrochemical, and polymer-composite routes. The synthesis method is therefore pivotal in defining the final electrochemical performance of Nd_2_O_3_ electrodes. Conventional strategies such as hydrothermal, solvothermal, combustion, electrodeposition, and solid-state synthesis have been widely used but often suffer from drawbacks, including high-temperature requirements, long reaction times, complex equipment setups, and limited reproducibility [26,27]. In contrast, the sol–gel process provides molecular-level mixing, precise stoichiometric control, and the ability to finely tune particle size, porosity, and crystallinity through simple adjustments in precursor concentration and reaction conditions. This tunability is of particular importance because the nucleation and growth dynamics during sol–gel processing directly influence surface area, ion-accessible porosity, and electrochemical kinetics, thereby dictating the overall charge storage capability. Moreover, sol–gel synthesis offers additional advantages of scalability, low energy consumption, and environmental compatibility, making it highly suitable for the fabrication of next-generation electrode materials [28].

In this study, we employ the sol–gel method to synthesize Nd_2_O_3_ with systematically varied precursor concentrations in order to establish clear correlations between synthesis parameters, microstructural features, and electrochemical properties. Structural and morphological analyses are combined with comprehensive electrochemical evaluation in an asymmetric supercapacitor configuration, providing deep insights into the structure–property performance relationship of Nd_2_O_3_ electrodes. Additionally, recent studies have revealed that surface morphology itself can act as an effective “geometric dopant,” where nanoscale curvature, surface roughness, and hierarchical topography modify the local electronic environment and charge distribution without altering chemical composition. Such geometric doping effects can enhance active site density, facilitate charge carrier mobility, and improve overall electrochemical kinetics [29]. Considering these insights, this work further examines how morphological variations induced by precursor concentration influence the potential geometric doping behavior in Nd_2_O_3_, thereby contributing to its superior capacitive performance. This investigation not only advances the understanding of precursor-driven microstructural evolution in rare-earth oxides but also highlights the potential of sol–gel synthesis as a rational and scalable strategy for designing high-performance electrode materials for future energy storage technologies.

## 2. Results and Discussions

### 2.1. XRD Elucidation

**Figure 1:**
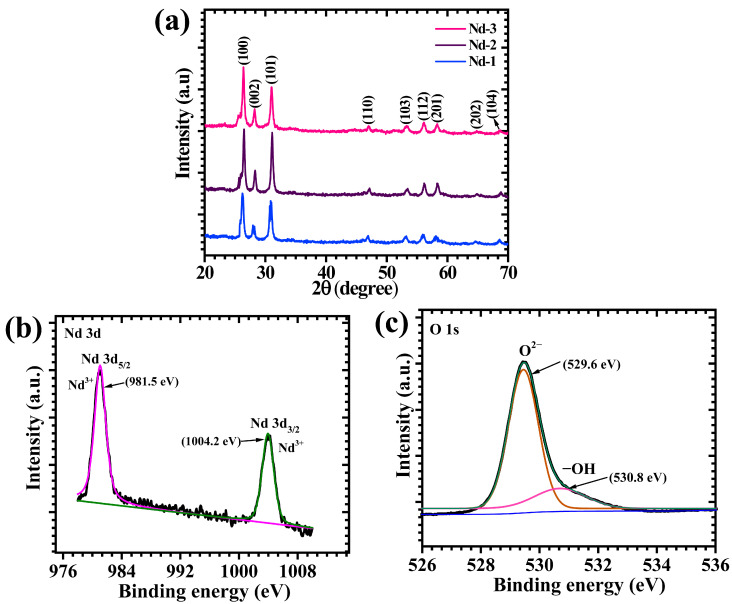
(**a**) XRD pattern of Nd-1, Nd-2, and Nd-3 electrodes, XPS spectra of (**b**) Nd 3d and (**c**) O 1s spectra of Nd-2 electrodes.

The X-ray diffraction (XRD) patterns of the Nd-1, Nd-2, and Nd-3 samples, synthesized by adjusting precursor concentrations, display clear distinctions in their crystal structure, as shown in Figure 1a. All three samples show characteristic diffraction peaks at 2θ values around 26.38°, 28.24°, 31.06°, 47.01°, 53.36°, 56.18°, 58.24°, 64.86°, and 68.45°, which correspond to the (100), (002), (101), (110), (103), (112), (201), (202), and (104) crystallographic planes of hexagonal Nd_2_O_3_, matching well the JCPDS card No. 00-041-1089. This confirms that each sample contains the hexagonal phase structure, successfully formed under the different synthesis conditions. As the precursor concentration increases from Nd-1 to Nd-3, the peaks become sharper and more intense, indicating that the crystallinity of the material improves with higher precursor amounts. This suggests more effective nucleation and growth of well-ordered crystals within the lattice, resulting in larger and more uniform crystallites. The slight variations in peak intensities and some minor peaks also reflect subtle differences in crystal orientation and growth dynamics influenced by synthesis parameters [30,31]. Such clear improvements in crystallinity are particularly important because they contribute to improved electrical conductivity and ion diffusion, which are critical for electrochemical performance. A well-ordered crystal lattice reduces defects and provides a better pathway for charge carriers, supporting faster and more efficient redox reactions. Moreover, quantitative XRD analysis was performed to evaluate the average crystallite sizes of all synthesized samples. The crystallite size D was estimated using the Scherrer Equation (1) [32]:

(1) $$D=\frac{K\lambda }{\beta cos\theta }$$

where K is the shape factor (0.9), λ is the X-ray wavelength, β is the FWHM in radians, and θ is the Bragg angle. The calculated average crystallite sizes are 152.1, 159.2, 171.7 nm for Nd-1, Nd-2, and Nd-3, respectively. While increased crystallinity correlates with decreased grain boundary density and could enhance conductivity, excessively large crystallites (Nd-3) lead to diminished active surface area for ion interaction and charge storage. This effect is well-recognized in the supercapacitor literature, where intermediate grain sizes often enable both fast charge/discharge kinetics and high capacitance by maximizing both electrical pathways and electroactive interface density. The results corroborate this paradigm: Nd-2 with moderate crystallinity and balanced grain size outperforms the highly crystalline Nd-3, which suffers from limited surface area and therefore reduced capacitance and rate performance [33,34]. In this context, the Nd-2 sample stands out, combining excellent crystallinity with an optimized nanostructure that enhances its electrochemical activity. This structural advantage underpins the superior charge storage capabilities and rate performance observed in the electrochemical tests, confirming Nd-2 as the most promising candidate among the series [22].

### 2.2. XPS Analysis

The surface electronic configuration and oxidation states of the optimized Nd-2 electrode were systematically investigated using high-resolution X-ray photoelectron spectroscopy (XPS). As illustrated in Figure 1b, the Nd 3d spectrum reveals two distinctly resolved doublets assigned to Nd 3d_5/2_ and Nd 3d_3/2_, positioned at binding energies of 981.5 eV and 1004.2 eV, respectively. These spectral features are analytic of Nd^3+^ species, unequivocally verifying that Nd predominantly exists in the trivalent state within the Nd_2_O_3_ framework with clear spin–orbit splitting (22.7 eV). These results are I excellent accordance with the reported Nd_2_O_3_ data [35]. The sharp and symmetric line shapes, accompanied by the absence of pronounced satellite structures, further attest to the high phase purity of the material and the negligible contribution from mixed-valence states or defect-induced electronic levels. This observation is in excellent accord with crystallographic data derived from XRD, collectively substantiating the successful stabilization of stoichiometric Nd_2_O_3_ in the Nd-2 electrode [36]. The O 1s spectrum of Nd-2, shown in Figure 1c, provides additional insight into the surface chemical environment. Deconvolution of the spectrum yields two principal components. The dominant peak located at 529.6 eV corresponds to lattice oxygen (O^2−^), reflective of the strong ionic interactions between Nd^3+^ and O^2−^ in the crystalline matrix [37,38]. A secondary peak at 530.8 eV is attributed to surface hydroxyl (-OH) groups, typically originating from adsorbed oxygen-related surface defects. The relative intensity of the hydroxyl component indicates the presence of surface defects or physiosorbed species, which can enhance surface wettability and provide additional electrochemically active sites. The XPS results confirm that the Nd-2 electrode contains Nd in the stable +3 oxidation state and oxygen species in both lattice and surface hydroxyl environments. These chemical states are expected to contribute significantly to the electrochemical performance of the Nd-2 electrode by promoting charge transfer and providing abundant surface-active sites [39,40].

### 2.3. Morphological and Elemental Composition

**Figure 2:**
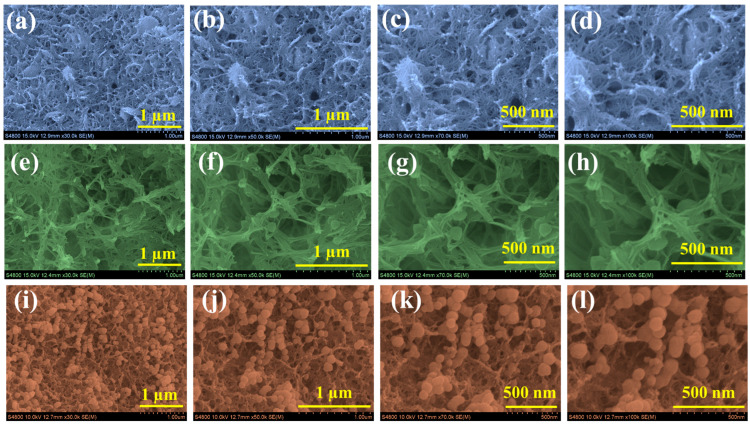
FE-SEM images of (**a**–**d**) Nd-1 (**e**–**h**) Nd-2, and (**i**–**l**) Nd-3 samples at different magnifications (100 k–30 k).

Figure 2 shows FESEM images of the Nd-1, Nd-2, and Nd-3 samples, revealing how changing precursor concentration influences the surface morphology. The Nd-1 sample (Figure 2a–d) exhibits a network primarily composed of thin, intertwined fibrous structures forming a relatively dense and compact mesh. Upon higher magnifications, these fibrous strands display scattered, small granule-like particles loosely attached to their surfaces. The granules appear sparsely distributed, and the fibers themselves show variable thickness, resulting in a moderately rough surface texture interspersed with accessible voids. In contrast, the Nd-2 sample (Figure 2e–h) reveals a more pronounced and extensively interconnected fibrous framework. This network forms a spacious, three-dimensional architecture characterized by well-defined and regular spacing among fibers. Notably, the fiber surfaces are heavily adorned with numerous fine granules, enhancing surface roughness and likely increasing the density of electrochemically active sites. Beyond the increased surface area, this hierarchical fiber-plus-granule morphology can induce geometric doping effects, where local variations in surface curvature and nanoscale elevations modify the electronic environment of Nd_2_O_3_. These effects can enhance local charge density, improve electron transport pathways, and facilitate ion adsorption, providing an additional structural contribution to the superior electrochemical performance for Nd-2. The combination of an open porous scaffold uniformly coated with abundant granular structures supports improved electrolyte infiltration, enhanced ion diffusion, and efficient charge transfer. For the Nd-3 sample (Figure 2i–l), the morphology shifts notably towards larger and denser granules, which overwhelmingly cover and obscure much of the original fibrous network. Although the fibers remain discernible at the base, their surface is dominated by tightly agglomerated spherical granules. This denser morphology reduces the effective surface area accessible to the electrolyte and likely impedes ion transport, and diminishes the geometric doping effects, which can explain the relatively lower electrochemical activity observed for this sample. All three samples share a fibrous foundational morphology, the extent and nature of granular growth on the fibers increase with higher precursor concentrations. The Nd-2 sample exemplifies an optimal morphological balance, where a well-connected fibrous network is uniformly decorated with fine and evenly distributed granules, maximizing available active sites and geometric doping contributions without compromising porosity. This hierarchical fiber-plus-granule structure effectively facilitates charge storage and transport, underpinning the excellent electrochemical properties demonstrated by Nd-2 [41,42,43].

**Figure 3:**
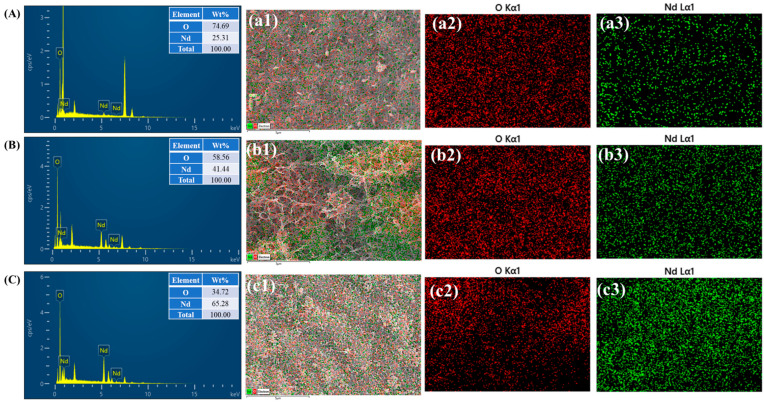
EDS spectra, corresponding SEM overlays, and elemental mapping images of (**A**–**a3**) Nd-1, (**B**–**b3**) Nd-2, and (**C**–**c3**) Nd-2 electrodes showing uniform distribution of Nd, and O elements.

Elemental composition analysis of the Nd-1, Nd-2, and Nd-3 electrode samples was conducted using energy-dispersive X-ray spectroscopy (EDS). The spectra shown in Figure 3A–C confirm the presence of Nd and oxygen as the primary elements, verifying the formation of Nd_2_O_3_ phases in all samples. The insets display the corresponding elemental weight percentages, indicating consistent stoichiometric ratios across the series. To further elucidate the spatial distribution, EDS elemental mapping was performed, as shown in Figure 3(a1–c3), along with FESEM overlay image. These maps demonstrate a uniform and homogeneous distribution of Nd and oxygen throughout the electrode surfaces for all samples. Such elemental uniformity supports the structural integrity and reliable synthesis of the Nd_2_O_3_ electrodes, which is crucial for their consistent electrochemical performance. In particular, the well-dispersed elemental pattern in Nd-2 aligns with its optimized morphology and enhanced capacitive behavior observed in electrochemical testing.

### 2.4. Electrochemical Analysis

**Figure 4:**
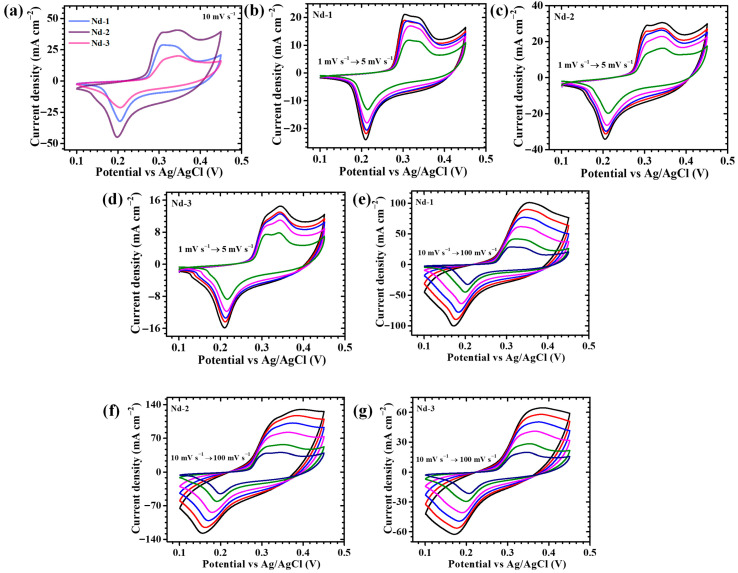
Cyclic voltammetry of (**a**) Nd-1, Nd-2, and Nd-3 samples were recorded at a scan rate of 10 mV/s, in a potential window from 0.1 to 0.45 V, cyclic voltammetry of (**b**) Nd-1, (**c**) Nd-2, (**d**) Nd-3, sample at different scan rates (1–5 mV s^−^^1^), (**e**) Nd-1, (**f**) Nd-2, (**g**) Nd-3, sample at different scan rates (10–100 mV s^−1^).

The electrochemical investigations of nanostructured Nd_2_O_3_ electrodes demonstrate a remarkable correlation between their engineered morphology and inherent redox properties. Cyclic voltammetry (CV) curves recorded at a scan rate of 10 mV s^−1^ within the potential window of 0.1–0.45 V (vs. Ag/AgCl) present highly distinct anodic (~0.32 V) and cathodic (~0.20 V) peaks, both sharp and symmetrical in nature (Figure 4a). These redox features are indicative of strongly reversible pseudocapacitive charge storage, primarily arising from fast surface faradaic reactions involving hydroxide ion adsorption/desorption and proton-coupled surface processes at Nd^3+^ sites. The continual engagement of neodymium cationic sites with hydroxide ions in the alkaline electrolyte ensures a significant faradaic contribution, supporting efficient charge storage kinetics [44,45]. Within the studied electrode series, the composition synthesized with a 3 mM Nd precursor (*Nd-2*) demonstrates the most distinguished electrochemical performance. This is evident from the enlarged CV area and strengthened redox currents, collectively reflecting a large capacitive response, faster charge propagation, and substantially diminished interfacial resistance. Such outcomes reveal the decisive impact of regulating precursor concentration in the synthetic route. In this optimal condition, the Nd_2_O_3_ matrix evolves into a highly integrated and seamless architecture that maximizes electroactive exposure and promotes rapid OH^−^ mobility, strengthening electrode–electrolyte interactions. Additionally, the nearly symmetric anodic/cathodic patterns confirm the quasi-reversibility of the reaction, implying minimal polarization and long-term stability over cycling. The governing electrochemical storage can be represented through surface-mediated faradaic transformations, schematically expressed as (2), (3) [39]:

(2) $${Nd}_{2}O_{3}+H_{2}O⇌\;2NdOOH$$

(3) $$2NdOOH+{OH}^{-}⇌\;Nd{\left(OH\right)}_{2}+e^{-}$$

These processes encapsulate the reversible oxidation–reduction switching of Nd oxide via continuous intercalation and de-intercalation of hydroxide ions. The carefully optimized precursor molarity maximizes this cooperative redox activity, while simultaneously increasing the conductivity of the network and reinforcing structural durability against cycling-induced stress.

The evaluation of rate capability further clarifies kinetic reversibility. CV profiles collected at both lower sweep rates (1–5 mV s^−1^, Figure 4b–d) and higher regimes (10–100 mV s^−1^, Figure 4e–g) consistently exhibit broad, well-defined faradaic peaks across all samples. Their non-rectangular shape confirms pseudocapacitance as the dominant energy storage contribution, in contrast to electrostatic double-layer effects. The observation of intensified peaks at higher scan rates also underscores the rapid interfacial charge-transfer processes driven by rational nanostructural design. Among the three electrode variants, the Nd-2 sample again shows significant superiority. Its optimized structure achieves an ideal compromise of particle uniformity, dispersion, and surface accessibility, interpreting to stronger faradaic responses, wider CV curve envelopes, and accelerated ionic kinetics. Mechanistically, the excellence of Nd-2 arises from the 3 mM precursor concentration, which ensures a finely tuned balance between nucleation and growth. This equilibrium fosters the evolution of homogeneous, hierarchically porous frameworks decorated with accessible pathways, thereby facilitating rapid OH^−^ ingress and efficient electron transport. In contrast, the 1 mM sample suffers from insufficient nucleation, yielding unstable morphologies with poorly connected domains. Alternatively, the 5 mM system promotes uncontrolled grain overlap and surface overloading, which hinders electroactive accessibility and leads to sluggish kinetics. These impaired structural states limit redox participation and dampen capacitive performance [46]. Detailed CV analysis highlights that Nd-2 uniquely exhibits intensified redox currents and expanded capacitive windows, directly traceable to its interconnected porous-web morphology with well-resolved voids. This architecture vastly enhances electrolyte penetration, shortens ionic diffusion pathways, and unlocks a higher density of active centers. The cooperative synergy between optimized morphology and redox activity thus accelerates the charge–discharge mechanism, yielding a highly reversible and durable pseudocapacitive response.

**Figure 5:**
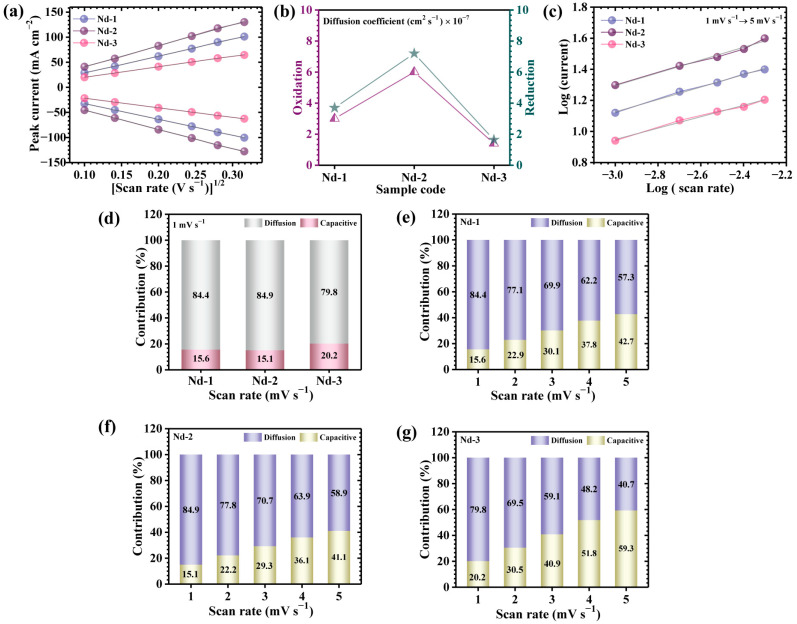
(**a**) Plot of peak current vs. (scan rate)^1/2^ of all samples for the diffusion coefficient, (**b**) plot of diffusion coefficient for all samples (**c**) Plot of *log*(*i*) against the *log*(*ϑ*) of all Nd_2_O_3_ samples, Plot of (**d**) Capacitive and diffusion-controlled processes of Nd_2_O_3_ electrodes at a scan rate of 1 mV s^−^^1^, Comparative evaluation of the capacitive and diffusion-controlled contributions of (**e**) Nd-1, (**f**) Nd-2 and (**g**) Nd-3 electrodes.

A comprehensive kinetic assessment of redox dynamics and ion transport behavior in Nd_2_O_3_-based electrodes was performed through CV over a wide range of scan rates. As depicted in Figure 5a, both anodic and cathodic peak currents (*i_p_*) exhibited a clear linear dependence on the square root of the scan rate (*v*^1/2^) across all investigated samples. The strong proportionality unequivocally confirms that the charge storage mechanism is predominantly governed by diffusion-controlled faradaic processes, characteristic of highly reversible redox interactions. To extract quantitative insights into ionic diffusion properties, the apparent diffusion coefficients (D) were evaluated using the Randles–Sevcik Equation (4) [47]:

(4) $$D^{1/2}=\frac{i_{p}}{2.69\times 10^{5}\times n^{3/2}\times A\times C\times v^{1/2}}$$

where *n* represents the electron transfer number (assumed as 1), *A* is the active surface area (1 cm^2^), *C* is the molar concentration of redox-active ions (2 M), and *v* corresponds to the scan rate. The calculated diffusion coefficients at 10 mV s^−^^1^ are tabulated in Table 1, with comparative graphical representation in Figure 5b. Among all electrode variations, Nd-2 consistently demonstrated the highest diffusion coefficient, signifying superior ionic transport kinetics and faster charge propagation pathways. This pronounced enhancement is intrinsically linked to its hierarchically engineered porous architecture, derived from the optimized 3 mM precursor concentration. The interconnected and open web-like morphology ensures unobstructed OH^−^ penetration, homogeneous distribution of electroactive centers, and enhanced accessibility to reaction interfaces, thereby accelerating both electron and ion flux. In contrast, electrodes synthesized from sub-optimal precursor concentrations displayed significant limitations. For Nd-1, insufficient nucleation led to restricted structural connectivity and a decline in the effective diffusion pathway, thereby. On the other hand, Nd-3 showed the lowest diffusion coefficient, resulting from excessive precursor content that induced particle agglomeration and surface encapsulation. Such morphological congestion minimizes accessible porosity, severely restricts electroactive site utilization, and hinders rapid ion transport, ultimately compromising the faradaic charge storage efficiency [48].

The fundamental charge storage characteristics of the Nd_2_O_3_ electrodes were further interrogated through quantitative power-law analysis, which describes the correlation between the peak current (*i_p_*) and the applied sweep rate (*v*) (5) [15]:

(5) $$i\;={av}^{b}$$

where the exponent b represents a critical descriptor of the underlying electrochemical mechanism. A value of b-0.5 signifies a diffusion-dominated faradaic regime, whereas values approaching unity indicate capacitive surface-limited storage. Log–log plots of current versus scan rate (Figure 5c) enabled accurate extraction of b-values through linear regression. For the Nd_2_O_3_ electrodes, the obtained values ranged narrowly between 0.36 and 0.42 (Table 1), thereby confirming that the governing charge storage originates primarily from ion-diffusion-controlled redox processes, with minor yet measurable contributions from capacitive interactions.

To refine mechanistic resolution between capacitive and diffusion-associated storage, the measured current response across variable potentials was deconvoluted according to the following kinetic expression (6) [49]:

(6) $$i\left(V\right)=k_{1}v{\;+\;k_{2}v}^{1/2}$$

In this formulation, the *k*_1_*v* term designates the non-faradaic capacitive contribution localized at the electrode–electrolyte interface, whereas the *k*_2_*v*^1/2^ term characterizes the diffusion-mediated charge transport through redox-active domains. By plotting *i(V)/v*^1/2^ vs. *v*^1/2^, values of *k*_1_ and *k*_2_ were accurately determined, enabling quantitative separation of the capacitive and diffusive charge contributions. On this basis, the overall stored charge can be mathematically expressed as (7) [49]:

(7) $$Q_{t}=Q_{s}+Q_{d}$$

where *Q_s_* represents interface-driven capacitive storage, and *Q_d_* reflects ion-diffusion-dominated faradaic storage. At a representative slow scan rate of 1 mV/s, the quantitative analysis revealed that diffusion-controlled contributions overwhelmingly dominated for all Nd_2_O_3_ electrodes, yielding fractional ratios of 15.6/84.4%, 15.1/84.9%, and 20.2/79.8%, for Nd-1, Nd-2, and Nd-3, respectively (Figure 5d). Among all the variants, the Nd-2 electrode displayed the most remarkable kinetic balance, with diffusion-controlled storage nearly reaching 84.9%, signifying exceptional ionic transport and accelerated reaction kinetics. This dominance can be mechanistically ascribed to its hierarchically organized porous network with interconnected void channels, ensuring unobstructed electrolyte accessibility and facilitating deep ion penetration into the electroactive matrix. Moreover, the evolution of charge storage dynamics as a function of increasing scan rate (1–5 mV/s) revealed a progressive increase in capacitive contributions across the entire electrode series. This behavior was consistently observed for Nd-1, Nd-2, and Nd-3 (Figure 5e–g). The shift toward capacitive dominance at higher scan rates arises from kinetic limitations of electrolyte ion penetration: accelerated sweep conditions constrain diffusion length, forcing surface-mediated capacitive processes to dominate. Nevertheless, Nd-2 sustains the highest fraction of diffusion-governed storage even under elevated scan rates, highlighting its morphological advantage in mitigating mass-transfer constraints. Collectively, these findings underscore the pivotal role of optimizing precursor concentration in governing the mechanistic interplay of charge storage. Specifically, the 3 mM Nd-2 electrode achieves an optimal state where porous alignment, electroactive accessibility, and electron–ion transport pathways are maximized, enabling diffusion-controlled pseudocapacitance to act as the dominant charge storage mechanism. This optimized balance directly translates into superior electrochemical kinetics and enhanced device efficiency when compared against lower (Nd-1) and higher (Nd-3) precursor concentrations [50,51].

**Figure 6:**
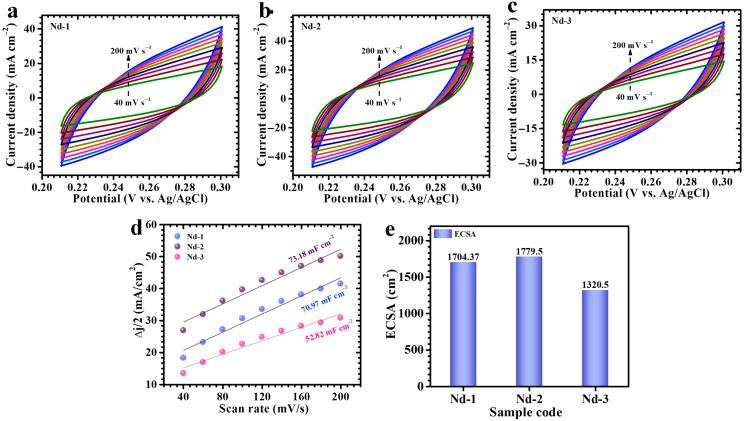
(**a**–**c**) CV measurements at various scan rates within a non-faradaic region for all electrodes, (**d**) Plot of resulting current variations at different scan rates for assessments of double-layer capacitance (C_dl_), and (**e**) The computed ECSA values for the Nd-1, Nd-2, and Nd-3 electrodes.

To assess the electrochemically active surface area (ECSA) of the Nd-1, Nd-2, and Nd-3 electrodes, *CV* was conducted at various scan rates confined to the non-faradaic region, as shown in Figure 6a–c. Utilizing this potential window ensured the measurement of pure capacitive current, eliminating interference from faradaic reactions. The resulting currents recorded at different scan rates were then used to evaluate the electrodes’ double-layer capacitance (Cdl), presented in Figure 6d. The ECSA was subsequently determined using the following relation (8) [52],

(8) $$ECSA=\frac{c_{dl}}{C_{s}}$$

where Cdl denotes the double-layer capacitance and Cs is the specific capacitance value attributed to the material (0.04 mF cm^−2^; in 1 M KOH). This approach offers a reliable means of quantifying the fraction of the electrode surface involved in charge storage, which is essential for understanding the energy storage behavior. Based on this method, the derived ECSA evaluations for Nd-1, Nd-2, and Nd-3 electrodes are approximately 1704.37, 1779.5, and 1320.5 cm^2^, respectively, as depicted in Figure 6e. The substantially higher ECSA of the Nd-2 sample indicates a highly porous and accessible surface, which supports effective ion transport and correlates directly with its improved electrochemical performance.

**Figure 7:**
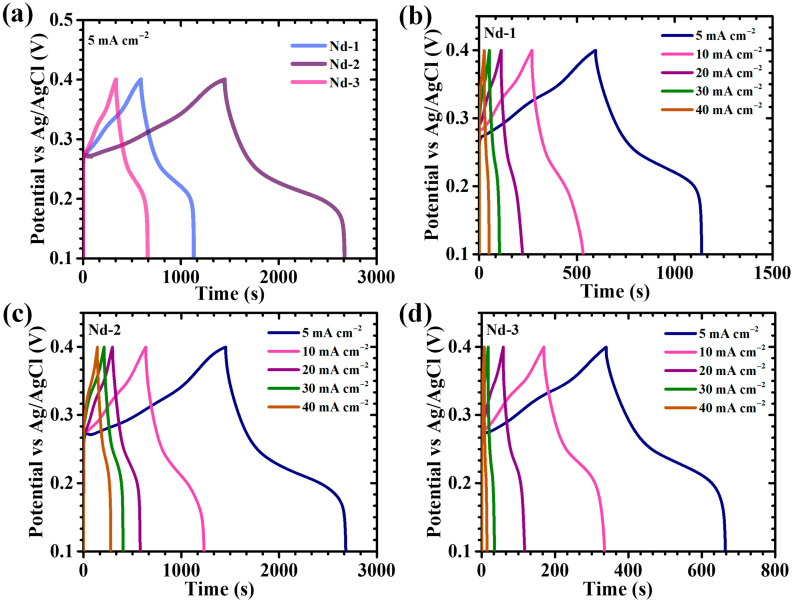
Galvanostatic charge–discharge curves of (**a**) Nd-1, Nd-2, and Nd-3 electrodes at 5 mA cm^−2^ current density, GCD plot of (**b**) Nd-1, (**c**) Nd-2 and (**d**) Nd-3 electrodes at various current densities.

**Figure 8:**
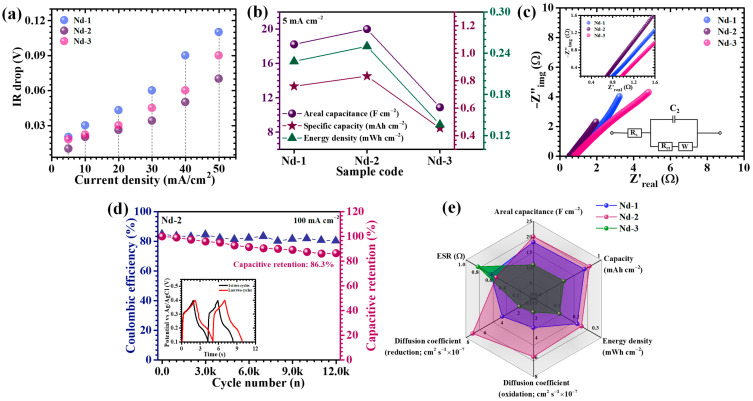
Plot of (**a**) IR drop trend, (**b**) energy storage parameters comparison, (**c**) Nyquist plots and zoomed-in high-frequency region, (**d**) long-term cycling performance of Nd-2 over 12,000 cycles, and (**e**) radar plot summarizing the multi-parameter electrochemical performance of Nd_2_O_3_ electrodes.

To elucidate the influence of precursor concentration on the electrochemical characteristics of Nd_2_O_3_ electrodes, galvanostatic charge–discharge (GCD) measurements were performed. Figure 7a illustrates the GCD profiles of the Nd-1, Nd-2, and Nd-3 electrodes, recorded at a current density of 5 mA/cm^2^ within a potential window of 0.1–0.4 V. All electrodes demonstrated nonlinear charge and discharge curves featuring distinct voltage plateaus, which are indicative of diffusion-limited faradaic processes characteristic of battery-type energy storage. Among the samples, Nd-2 exhibited the most pronounced nonlinear behavior characterized by a smooth and consistent discharge slope. This response signals pseudocapacitive storage dominated by reversible hydroxide ion intercalation and surface redox reactions. Moreover, the longer discharge duration of Nd-2 relative to Nd-1 and Nd-3 confirms its enhanced charge storage capacity, attributable to its finely controlled nanostructure. Further, GCD tests conducted across a range of current densities (5 to 40 mA/cm^2^, Figure 7b–d) corroborated the predominance of pseudocapacitive mechanisms. Discharge curves consistently preserved redox-associated voltage plateaus, complementing the CV data and underscoring the faradaic nature of charge storage. All electrodes exhibited near-perfect symmetry between charge and discharge segments, indicative of high coulombic efficiency, negligible polarization losses, and efficient ion transport kinetics. Notably, Nd-2 showed the lowest initial IR drop at discharge onset and sustained symmetrical charge–discharge profiles over the entire current density spectrum, reflecting its diminished internal resistance, superior electronic conductivity, and highly reversible redox transitions. IR drop analysis (Figure 8a) revealed a systematic reduction in IR drop magnitude with decreasing current density across all electrodes, reflecting mitigated resistive losses at slow cycling rates. Consistently, Nd-2 demonstrated the minimal IR drop over the full current range, indicative of optimized electrode–electrolyte interface kinetics and minimized bulk resistance.

Quantitative electrochemical parameters including areal capacitance (C_A_), energy density (ED), and power density (PD) were computed using equations specifically formulated for nonlinear pseudocapacitive GCD responses (9)–(11) [53,54]:

(9) $$C_{A}=\frac{I\times 2\times \int V\left(t\right)dt}{A\times {\left(∆V\right)}^{2}}$$

(10) $$ED=\frac{1}{2\times 3600}\;C_{A}\times {dV}^{2}$$

(11) $$PD=\frac{ED\times 3600}{T_{d}}$$

where *I* is the applied current, $\int_{0}^{T}V\left(t\right)dt$ is the integral of the discharge voltage over the time interval from t = 0 (start of discharge) to t = T (end of discharge), *A* is the electrode geometric area, Δ*V* denotes the potential window, and *T* the discharge duration. These calculations account for the intrinsic faradaic nonlinearity, ensuring an accurate representation of electrode performance. At 5 mA cm^−2^, the areal capacitances of Nd-1, Nd-2, and Nd-3 were determined as 18.22, 20, 10.89 F cm^−2^ respectively (Table 2, Figure 8b), with Nd-2 outperforming its peers significantly. This superior capacitance is ascribed to several synergistic factors: (i) formation of a porous high-surface-area nanostructure rich in electroactive sites, (ii) enhanced electrolyte penetration and shortened ion diffusion pathways enabled by a cross-linked web-like nanoparticulate framework, and (iii) minimized particle agglomeration and restacking resulting from precisely controlled precursor content. Such morphology preserves hierarchical porosity critical for rapid ion transport and accelerated surface redox kinetics [46,55]. The observed decline in capacitance and energy density with increasing current (Table 2) reflects common rate limitations, where restricted hydroxide ion diffusion at elevated scan rates limits faradaic access to inner active sites. Under high-rate conditions, surface-accessible pseudocapacitive responses dominate as bulk diffusion becomes impeded [56]. Despite this trend, Nd-2 retained 84.45% of its initial capacitance at 40 mA cm^−2^, attesting to its remarkable rate capability and structural durability under rigorous cycling. Together, the GCD results unequivocally demonstrate that the Nd-2 electrode features an architecturally optimized framework characterized by minimized internal resistance, extensive electroactive surface exposure, and rapid ion/electron transport. This tailored morphology is central to its superior charge storage capacity, enhanced rate performance, and electrochemical stability relative to Nd-1 and Nd-3 electrodes.

Electrochemical impedance spectroscopy (EIS) was utilized to probe the intrinsic charge transport and interfacial electrochemical behavior of the Nd_2_O_3_ electrodes. Nyquist plots, measured over a frequency range of 1 kHz to 0.1 Hz in 2 M KOH electrolyte (Figure 8c), offer crucial insights into the electrolyte resistance, interfacial charge transfer, and ion diffusion kinetics. Each Nyquist spectrum features a compressed semicircle at high frequencies followed by an inclined linear tail at lower frequencies, effectively segregating different electrochemical phenomena. The high-frequency intercept on the real impedance axis (Z′) corresponds to the equivalent series resistance (ESR), which includes contributions from ionic resistance of the electrolyte, inherent electrode material resistance, and the contact resistance at the electrode/electrolyte interface. The diameter of the semicircle reflects the charge transfer resistance, serving as a direct indicator of faradaic reaction kinetics at the electrode surface [57]. EIS data for the Nd-1, Nd-2, and Nd-3 electrodes were quantitatively analyzed using a classical Randles equivalent circuit consisting of series resistance (ESR), double-layer capacitance (C2), charge-transfer resistance (Rct), and Warburg diffusion element (W). Fitting was performed using EC Lab software version 11.40 (Table 3). The extracted parameter values are as follows: for Nd-1, ESR = 0.63 Ω, C2 = 0.3325 F, Rct = 1.332 Ω, and W = 1.891 Ω·s^−0.5^; for Nd-2, ESR = 0.557 Ω, C2 = 0.6372 F, Rct = 1.074 Ω, and W = 0.7125 Ω·s^−0.5^; and for Nd-3, ESR = 0.83 Ω, C2 = 0.2252 F, Rct = 1.717 Ω, and W = 2.919 Ω·s^−0.5^. Comparison of these results reveals that Nd-2 exhibits the lowest series resistance and the highest double-layer capacitance, indicating its superior electrolyte accessibility and rapid interfacial charge storage capability. The Warburg element, which reflects ion diffusion processes, is also smallest for Nd-2, indicating more facile ion transport. Collectively, this analysis demonstrates that carefully optimized synthesis parameters for Nd-2 lead to pronounced improvements in both charge-transfer and ion-diffusion processes, providing a mechanistic explanation for its superior electrochemical performance. This minimal series resistance, coupled with decreased charge transfer resistance, accelerates charge transport processes, enhancing rate capability and significantly improving power output efficiency. The superior electrochemical characteristics of the Nd-2 sample thus relate fundamentally to its optimized nanostructure, which facilitates efficient electron pathways and promotes fast ion diffusion at the electrode/electrolyte interface, as reflected by its promising impedance profile.

Long-term cycling durability at elevated current densities is a critical parameter in assessing the practical applicability of supercapacitor electrodes. To investigate this, the cycling stability of the optimized Nd-2 electrode was rigorously evaluated via continuous GCD testing at 100 mA/cm^2^ over 12,000 successive cycles. The evolution of capacitance retention and coulombic efficiency as functions of cycle number are depicted in Figure 8d. Initially, the electrode displayed a gradual enhancement in capacitance, which can be ascribed to electrochemical activation phenomena and progressive pore unblocking that facilitates deeper electrolyte infiltration. Following this activation phase, the electrode performance stabilized, exhibiting minimal capacity degradation throughout the extended cycling period. After 12,000 cycles, the Nd-2 electrode retained approximately 86.3% of its initial capacitance, corresponding to a nominal loss of 13.7%, thereby demonstrating pronounced long-term electrochemical stability. This longevity is underpinned by the structural resilience and chemical robustness of the Nd_2_O_3_ framework, which accommodates volumetric fluctuations inherent to recurrent ion insertion/extraction without succumbing to mechanical fracturing or active site degradation. The hierarchical architecture of Nd-2 effectively mitigates pulverization and preserves the integrity of redox-active domains essential for sustained charge storage. Equally noteworthy is the consistently high coulombic efficiency observed throughout cycling, maintaining a notable 80.16% even after 12,000 charge–discharge cycles. Such efficiency indicates marginal parasitic side reactions and efficient charge compensation during faradaic processes, reinforcing the electrode’s reversible electrochemical behavior. Several well-documented factors may contribute to this moderate CE and gradual capacity fading including, transition metal and rare-earth oxide supercapacitors are known to undergo partial irreversibility in redox cycling, leading to incomplete charge recovery and energy loss in each cycle. Further extended cycling at high current densities can induce progressive dissolution, or structural changes in the active material, resulting in decreased electronic/ionic conductivity and lower CE. With repeated operation, slow parasitic reactions (such as electrolyte decomposition, and formation of resistive surface films) can occur, further reducing charge efficiency and capacity. Reduced CE may also arise from ion intercalation/deintercalation kinetics, if a fraction of electrolyte ions become trapped in the lattice or sites become blocked, full discharge is impeded. Despite these challenges, the data demonstrate high long-term capacitive retention (86.3% after 12,000 cycles), indicating robust electrode integrity even as some efficiency losses accumulate. Overall, the combination of sustained capacitance retention and persistently high coulombic efficiency underscores the electrochemical robustness and operational reliability of the Nd-2 electrode.

The post-cycling surface morphology was examined using FESEM (Figure S1(a1,a2)), revealing a transition from relatively smooth, interconnected network to a rougher, more fragmented texture after cycling. This morphological evolution, which includes the emergence of cracks and slightly increased irregularity is attributed to redox-driven surface oxidation, electrolyte-induced modification, and mechanical stress from continuous ion insertion/extraction. Such changes are expected in electrodes and remain modest, indicating robust microstructural endurance despite prolonged use. Further, XRD pattern (Figure S1b) retains all major diffraction peaks characteristic of the hexagonal Nd_2_O_3_ (JCPDS No. 00-041-1089), with no detectable impurity phases or significant peak shifts, confirming the preservation of the primary crystal structure. Moderate changes, such as increased background noise and reductions in peak intensity, suggest subtle lattice strain and limited surface restructuring due to repeated cycling. EIS was conducted before and after extended cycling to monitor changes in interfacial and bulk resistance. The Nyquist plots (Figure S1c) reveal a minor increase in ESR (0.78 Ω) after long-term cycling, suggesting the electrode maintains efficient charge transport pathways and interfacial stability throughout the cycling process. This gradual resistance evolution is consistent with minor surface reconstruction or limited electrolyte decomposition, confirming the overall durability of the electrode architecture under repetitive operation.

Figure 8e presents a radar chart that succinctly captures and compares multiple pivotal electrochemical parameters which including areal capacitance, specific capacity, energy density, ionic diffusion coefficient, and ESR for Nd-1, Nd-2, and Nd-3 electrodes. This multidimensional visualization distinctly highlights the superior and well-balanced electrochemical attributes of the Nd-2 sample. The comprehensive spatial coverage across all metrics reflects the successful integration of high charge storage capability, expedited ion transport kinetics, and reduced internal resistive losses, unified within a single, meticulously optimized electrode design. Such balanced performance underscores the efficacy of the structural and compositional tuning strategies employed to tailor Nd-2 electrode architecture for enhanced energy storage applications.

### 2.5. Electrochemical Performance of Asymmetric Supercapacitor Device

**Figure 9:**
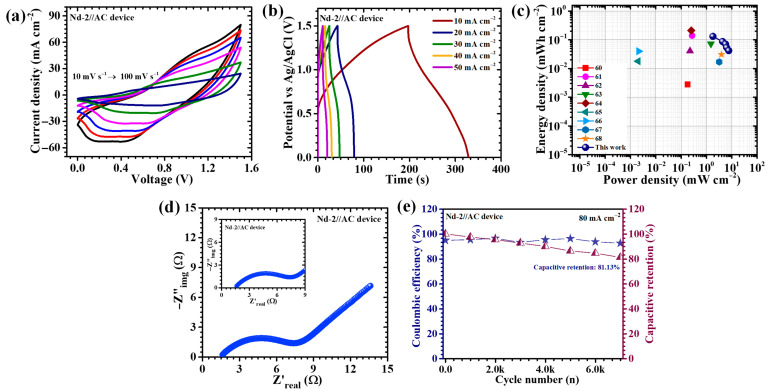
Electrochemical performance of the asymmetric supercapacitor device (Nd-2//AC): (**a**) CV curves at 0–1.5 V of the asymmetric supercapacitor device, (**b**) GCD curves at different current densities (10–50 mA cm^−2^), (**c**) Ragone plot of the device, (**d**) Nyquist plot, and (**e**) long-term cycling performance over 7000 cycles.

To bridge the gap between laboratory characterization and real-world usability, an asymmetric pouch-type supercapacitor device (APSD) was assembled using the best-performing Nd-2 electrode as the positive terminal and activated carbon (AC, acetylene black) as the negative terminal. The AC electrode was chosen due to its reliable electric double-layer capacitance, which complements the faradaic activity of Nd_2_O_3_. Both active materials were coated on NF current collectors to ensure excellent conductivity and mechanical stability. For AC electrode, we used acetylene black as the conductive carbon additive, polyvinylidene fluoride as the binder, and N-methyl-2-pyrrolidone as the solvent in preparing the electrode slurry. The slurry was homogenized to ensure uniform dispersion of the components, then coated on the current collector and dried under controlled conditions (60 °C for 12 h) to remove solvent completely. The device employed a 2 M KOH aqueous solution as the electrolyte, with filter paper acting as both separator and electrolyte reservoir. The pouch was carefully sealed to protect it from external contamination or humidity. The electrochemical performance was thoroughly investigated using CV, GCD, and EIS techniques. CV tests (Figure 9a) performed over scan rates from 10 to 100 mV/s revealed quasi-rectangular profiles decorated with distinct redox peaks. This combination reflects the dual contribution of surface capacitive storage from the AC electrode and diffusion-driven pseudocapacitance from Nd_2_O_3_. Remarkably, the APSD remained stable up to a working potential of 1.5 V, which is quite high for an aqueous system. This wide potential window stems from the strong synergy between the high redox activity of Nd_2_O_3_ and the large accessible surface area of AC. The GCD measurements (Figure 9b) reinforced these findings. Non-linear charge–discharge traces characteristic of pseudocapacitive processes were observed. At a current density of 10 mA cm^−2^, the device delivered a high areal capacitance of 424 mF cm^−2^, combined with an energy density of 0.132 mWh cm^−2^ and a power density of 1.8 mW cm^−2^. The energy–power density relationship of the fabricated asymmetric supercapacitor device based on the Nd-2 electrode is illustrated in the Ragone plot (Figure 9c) [58,59,60,61,62,63,64,65,66]. The Navy-blue symbols represent the performance metrics achieved in this work, while the other colored symbols correspond to previously reported supercapacitor systems from literature. Notably, the device in this study demonstrates a superior combination of energy and power densities, with values reaching up to 0.132 mWh cm^−2^ at a power density of 1.80 mWcm^−2^. Even at higher power densities (7.58 mWcm^−2^), the energy density remains well-retained, highlighting the device’s excellent rate capability and fast charge–discharge characteristics. Compared to other reports, the Nd-2//AC device exhibits outstanding electrochemical performance, outperforming many previously published systems in both energy and power metrics. This balance between high energy storage and fast power delivery indicates the strong potential of the device for practical applications. Impedance studies gave more insight into its internal properties (Figure 9c). The Nyquist plots showed a small semicircle in the high-frequency region which evidence of low charge-transfer resistance followed by a steep straight line at low frequencies, typical of efficient capacitive behavior. The ESR of just 1.62 Ω indicates excellent conductivity and easy ion movement within the electrode structures. This is directly linked to the optimized porous arrangement of the Nd-2 electrode, which ensures continuous electron pathways and smooth electrolyte penetration. Finally, the durability of the device was evaluated under continuous cycling at 80 mA cm^−2^ for 7000 cycles (Figure 9d). Notably, the APSD retained 81.13% of its initial capacitance while maintaining a coulombic efficiency of 92.49%. Such robustness can be credited to the stable nanosheet-based architecture of Nd-2, capable of withstanding repeated redox cycling without major structural breakdown. Altogether, these results show that the Nd-2//AC asymmetric device achieves a compelling combination of broad operating voltage, high capacitance, low resistance, and long-term cycling stability. These qualities make Nd_2_O_3_ electrodes, especially at the optimized precursor concentration, highly promising for next-generation supercapacitors intended for flexible and portable energy storage technologies.

## 3. Conclusions

In this work, we demonstrated that the electrochemical performance of Nd_2_O_3_ electrodes is strongly governed by precursor concentration during sol–gel synthesis. The 3 mM precursor sample (Nd-2) exhibited superior crystallinity, a well-defined hexagonal phase, and a hierarchically interconnected fibrous–granular morphology, which collectively enhanced ion diffusion and electron transport. Electrochemical evaluation revealed that Nd-2 achieved an areal capacitance of 20 F cm^−2^, with diffusion-controlled pseudocapacitance contributing ~84.9% and excellent cycling stability of 86.3% retention over 12,000 cycles. When incorporated into an asymmetric supercapacitor with activated carbon, the device delivered a high energy density of 0.132 mWh cm^−2^, power density of 1.8 mW cm^−2^, and retained 81.1% capacitance over 7000 cycles, demonstrating remarkable operational durability. These findings underscore the critical influence of precursor-driven microstructural tuning in rare-earth oxide electrodes and highlight the potential of Nd_2_O_3_ as a high-performance pseudocapacitive material. The study establishes a clear structure–property performance relationship, providing a rational framework for designing next-generation energy storage devices with optimized morphology, enhanced ion/electron transport, and long-term stability.

## 4. Materials and Methods

### 4.1. Sol–Gel Synthesis of Nd_2_O_3_ with Varied Precursor Concentrations and Electrode Fabrication

**Figure 10:**
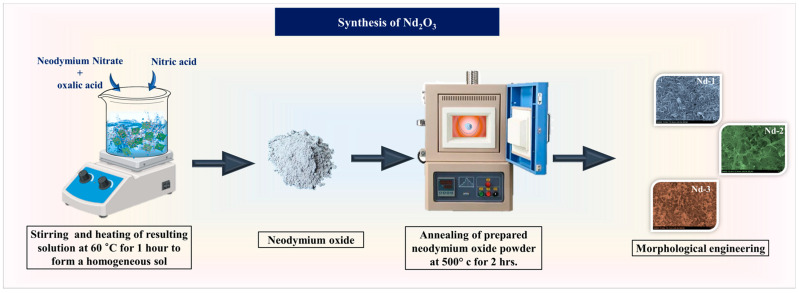
Intuitive schematic illustration of the synthetic pathway for the fabrication of Nd_2_O_3_ electrodes.

Nd_2_O_3_ was synthesized through a sol–gel method in which the precursor concentration was systematically varied to study its effect on structural and electrochemical properties. Neodymium (III) nitrate hexahydrate (Nd(NO_3_)_3_·6H_2_O, 99.9%, Sigma-Aldrich, St. Louis, MO, USA) was used as the neodymium (Nd) source and citric acid (C_6_H_8_O_7_, 99.5%, Sigma-Aldrich, St. Louis, MO, USA) served as a chelating agent to regulate gel formation. Absolute ethanol and deionized water were used as solvents, and dilute nitric acid (HNO_3_; 70%, Sigma-Aldrich, St. Louis, MO, USA) was added to maintain the solution pH between 2 and 3. Three precursor amounts, 1 mmol, 3 mmol, and 5 mmol, were used to prepare the sols. In a typical procedure, the required amount of Nd(NO_3_)_3_·6H_2_O was dissolved in deionized water under magnetic stirring until a clear solution was obtained. Citric acid dissolved in ethanol was added dropwise to the Nd solution at a molar ratio of 2:1 to achieve complete complexation. The resulting mixture was stirred at 60 °C for 1 h to form a homogeneous sol and then gradually heated to 80–90 °C to form a viscous gel. The gel was aged at 80 °C for 12 h to remove residual solvent and stabilize the polymeric network. The dried gels were subjected to a two-step calcination procedure to obtain crystalline Nd_2_O_3_. The gels were first heated to 300 °C at a rate of 2 °C/min and held for 1 h to remove organic residues and nitrate species. The temperature was then increased to 500 °C at a rate of 5 °C min^−1^ and maintained for 2 h to achieve complete crystallization. After natural cooling to room temperature, the calcined powders were ground to obtain fine and uniform particles. The synthetic pathway for the Nd_2_O_3_ is comprehensively illustrated in Figure 10, capturing the progressive stages of material assembly.

For electrode fabrication, the as-prepared Nd_2_O_3_ powders were mixed with carbon black (acetylene carbon) and polyvinylidene fluoride binder in a weight ratio of 80:10:10 using N-methyl-2-pyrrolidone to form a homogeneous slurry. Nickel foam (NF) substrates were cleaned by sonication in 1 M hydrochloric acid for 10 min, rinsed with deionized water and ethanol, and dried at 60 °C for 12 h. The resulting slurry (1 mg/cm^2^) was carefully applied to Ni-foam (1 cm^2^), which served as the electrode substrate due to its excellent electrical conductivity and porous architecture, allowing for efficient charge transport. and dried under vacuum at 80 °C for 12 h. The electrodes prepared with 1 mmol, 3 mmol, and 5 mmol of Nd (NO_3_)_3_·6H_2_O were designated as Nd-1, Nd-2, and Nd-3, respectively.

### 4.2. Sample Characterization and Electrochemical Measurements

The phase composition and crystallinity of the Nd_2_O_3_ electrodes were examined using XRD (PANalytical, Cu-Kα radiation), which allowed clear identification of crystalline phases and assessment of structural integrity. The surface morphology and microstructure were studied with FE-SEM (S-4800, HITACHI, Tokyo, Japan) coupled with energy-dispersive X-ray spectroscopy (EDS) to evaluate elemental distribution. Prior to imaging, a thin layer of platinum was sputtered onto the electrode surfaces to minimize charging effects. FE-SEM images revealed the detailed surface textures and particle arrangements, while EDS confirmed the presence and uniform dispersion of Nd and oxygen in the samples. XPS (K-Alpha, Thermo Scientific, UK) was employed to investigate the surface chemical states and oxidation states of Nd and O, providing insights into the material’s electronic structure. Electrochemical performance of the electrodes was evaluated using a Biologic WBCS3000 battery cycler in a three-electrode configuration. The Nd_2_O_3_-coated NF served as the working electrode, platinum as the counter electrode, and Ag/AgCl as the reference electrode. 2 M aqueous KOH solution was used as the electrolyte to study the capacitive behavior, charge–discharge profiles, rate capability, and long-term cycling stability.

## Figures and Tables

**Table 1:** Calculated Diffusion coefficient, b-values, and series resistance values of Nd-1, Nd-2, and Nd-3 electrodes.

Sample Code	Diffusion Coefficient (cm^2^ s^−1^) × 10^−7^		b-Value	ESR (Ω)	Rct (Ω)
	Oxidation	Reduction			
Nd-1	3	3.7	0.4	0.63	1.332
Nd-2	6	7.2	0.42	0.55	1.074
Nd-3	1.4	1.64	0.36	0.82	1.717

**Table 2:** Comparison of calculated areal capacitance, specific capacity, energy density, and power density values of Nd_2_O_3_ electrodes.

Sample Code	I (mA/cm^2^)	Areal Capacitance C_A_ (F cm^−2^)	Capacity (mAh cm^−2^)	Energy Density ED (mWh cm^−2^)	Power Density PD (mW cm^−2^)
**Nd-1**	5	18.222	0.759	0.228	0.74
	10	14.222	0.593	0.178	1.53
	20	8.933	0.372	0.112	2.45
	30	6.756	0.281	0.084	3.76
	40	4	0.167	0.05	5.85
**Nd-2**	5	20	0.833	0.25	1.27
	10	19.556	0.815	0.244	3.07
	20	19.111	0.796	0.239	4.66
	30	18.556	0.773	0.232	5.57
	40	16.889	0.704	0.211	6.1
**Nd-3**	5	10.889	0.454	0.136	0.75
	10	9.889	0.412	0.124	1.5
	20	5.333	0.222	0.067	2.84
	30	1.956	0.081	0.024	3.83
	40	1	0.042	0.013	4.09

**Table 3:** Electrochemical performance of the Nd-2//AC asymmetric pouch-type supercapacitor device at various current densities.

Sample Code	I (mA)	CA (mF cm^−2^)	C (mAh cm^−2^)	ED (mWh cm^−2^)	PD (mW cm^−2^)
**Nd-2//AC device**	10	424	0.088	0.132	1.8
	20	277	0.058	0.087	4.33
	30	227	0.047	0.071	5.67
	40	172	0.036	0.054	6.26
	50	133	0.028	0.042	7.58

## Data Availability

The data presented in this study are available on request from the corresponding author.

## Supplementary Materials

The following supporting information can be downloaded at https://www.mdpi.com/article/10.3390/gels11110883/s1, Figure S1: (a1 and a2) FESEM images at different magnifications illustrating the morphological evolution of the electrode after long-term cycling, (b) XRD pattern of the Nd-2 electrode after 12,000 charge–discharge cycles, and (c) Nyquist plot of Nd-2 electrode before and after stability test.
