# Supplementary material for: Precursor Concentration-Dependent Sol–Gel Dynamics in Neodymium Oxide: From Gel Framework to Electrochemical Functionality in Asymmetric Supercapacitors
Source: Gels. 2025 Nov 3;11(11):883. doi: 10.3390/gels11110883 (PMC12652413; doi:10.3390/gels11110883)
Supplement: Supplementary file 1 [file gels-11-00883-s001.zip › gels-3938151-supplementary.pdf]

Supplementary Information

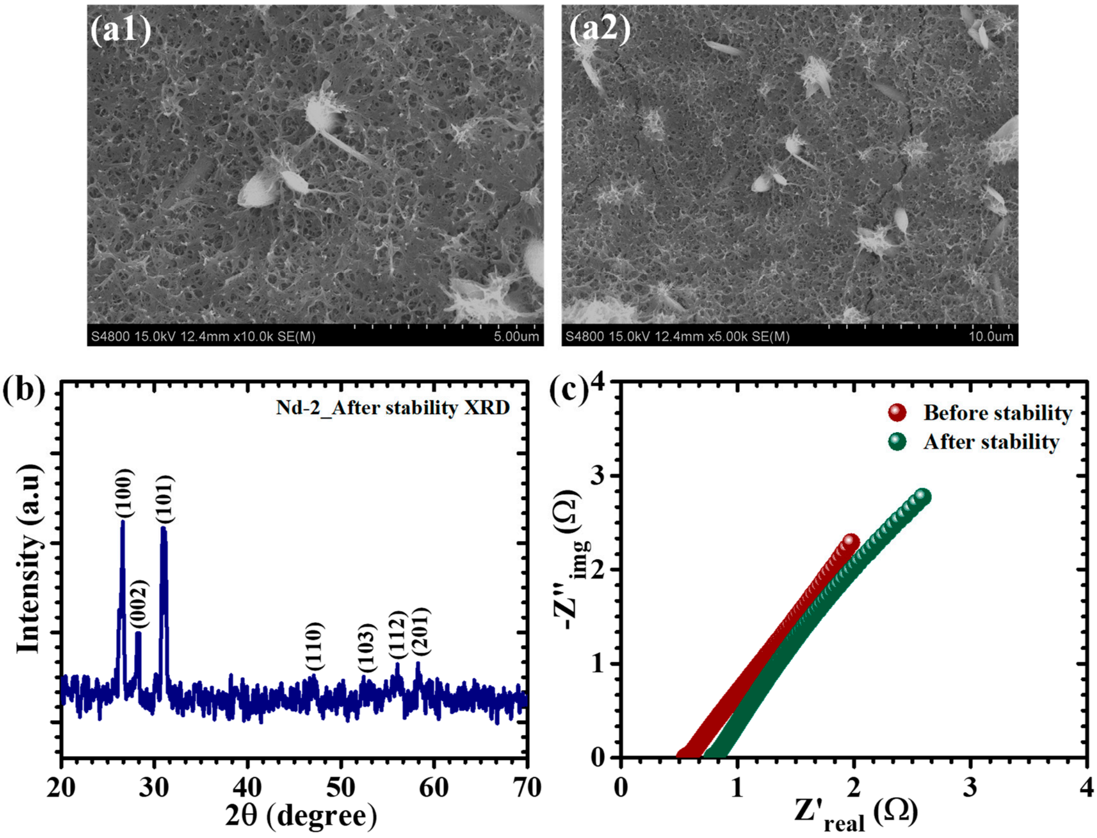

Figure S1: (a1 and a2) FESEM images at different magnifications illustrating the morphological evolution of the electrode after long-term cycling, (b) XRD pattern of the Nd-2 electrode after 12,000 charge–discharge cycles, and (c) Nyquist plot of Nd-2 electrode before and after stability test.
